# Peroneal tendoscopy – more than just a solitary procedure: case-series

**DOI:** 10.3325/cmj.2015.56.57

**Published:** 2015-02

**Authors:** Ivan Bojanić, Damjan Dimnjaković, Ivan Bohaček, Tomislav Smoljanović

**Affiliations:** Department of Orthopaedic Surgery, University Hospital Center Zagreb, School of Medicine, University of Zagreb, Zagreb, Croatia

## Abstract

This study presents a series of 13 patients who underwent peroneal tendoscopy as a solitary or accessory procedure at our department in 2013. Patients were clinically diagnosed with peroneal tendons disorders and underwent an additional radiological assessment. Peroneal tendoscopy was carried out in a standard manner before any other arthroscopic or open procedure. Postoperative management depended on the type of pathology. We found 3 peroneus brevis tendon partial tears, 4 cases of a low-lying peroneus brevis muscle belly, 5 cases of tenosynovitis, and 1 case of an intrasheath peroneal tendon subluxation. In 5 patients peroneal tendoscopy was performed as a solitary procedure and in 8 patients as an accessory procedure – together with anterior or posterior ankle arthroscopy, combined posterior and anterior ankle arthroscopy, or open surgery. Both as a solitary and accessory procedure, peroneal tendoscopy was safe and successful, ie, all patients were without any symptoms at one-year follow-up. Our series of patients showed that peroneal tendoscopy can be used both as an independent procedure as well as a valuable accessory procedure.

Peroneal tendoscopy or “endoscopy of the peroneal tendon sheath” (1) allows visualization of the peroneal tendons from the myotendinous junction to the peroneal tubercle, while preserving soft anatomical structures and providing a dynamic evaluation of their movement inside the sheath. Peroneal tendoscopy was first described by Van Dijk et al in 1998 (2), but it was rather slowly accepted by the orthopaedic community. Only in the last couple of years has the number of performed peroneal tendoscopies notably increased (1,3-5). Tendoscopy has been additionally popularized and used for evaluation and management of various pathologic conditions of other tendons around the ankle, most frequently for the posterior tibial tendon and Achilles tendon (6-9).

The peroneal tendons are situated subcutaneously along the lateral wall of the calcaneus and posterolateral aspect of the fibula. These tendons are easily accessible, which makes them good candidates for tendoscopic treatment of peroneal tendons disorders. Such disorders are frequently clinically expressed as posterolateral ankle pain and include a wide variety of disorders ranging from tenosynovitis, tendon dislocation, and subluxation to peroneal tendon rupture (whether partial or complete) (1,10-13). These conditions often occur combined with other symptoms of intra- or extra-articular pathology of the ankle such as lateral ankle instability, distal fibula fractures, anterior or posterior impingement of the ankle, chondral or osteochondral lesions of the talus, or subtalar malalignment (such as calcaneus varus) (1,10-13).

Peroneal tendoscopy is commonly used as an independent, ie, solitary procedure (2,5,11), but there are no literature findings on its use as an accessory procedure. We would like to present our series of 13 patients who underwent peroneal tendoscopy as a solitary or accessory procedure, showing that this procedure may have a dual role in the orthopaedic armamentarium – as a solitary procedure for certain indications, but also as a valuable accessory procedure to some other arthroscopic or open procedures dealing with intra- or extra-articular pathology in or around the ankle.

## Patients

Thirteen patients, 6 men and 7 women, with persistent posterolateral ankle pain underwent peroneal tendoscopy between January and December 2013. All the diagnostic and surgical procedures were performed at our department. Institutional review board approval was obtained together with patients' informed consents. All surgical procedures were performed by the same surgeon.

The patients' average age was 32 (range, 13 to 58) years at the time of surgery. All patients were clinically diagnosed with peroneal tendons disorders and underwent conventional radiographic, ultrasonographic, and magnetic resonance imaging assessment. In 5 patients peroneal tendoscopy was performed as an independent procedure and in 8 patients as an accessory procedure.

Peroneal tendoscopy was carried out in a standard manner before any other arthroscopic or open procedures in order to prevent fluid extravasation at the lateral side of the ankle. Under spinal anesthesia, patients were placed in the lateral decubitus position, with the operative side facing up. A thigh tourniquet was used during the whole procedure. Support was placed under the affected leg thus providing free intraoperative manipulation of the ankle. A 4.5-mm 30-degree arthroscope with a gravity irrigation system and basic standard arthroscopic instruments was regularly used. Standard portals described by Van Dijk and Kort (2) were created using a “nick and spread” technique in order to minimize the risk of injury to the sural or superficial peroneal nerve. The distal portal, located 1.5 to 2 cm distal to the tip of the fibula, was created first. The proximal portal, located 2 to 2.5 cm proximal to the tip of the fibula and 5 mm posterior to the fibula was made under direct vision by placing an intramuscular needle. After complete tendoscopic exploration, adequate tendoscopic treatment was performed. At the end of tendoscopic procedure, the portals were sutured. No suction drains were used. If needed, the patient was turned into desired position and prepared for an additional procedure, leaving all the instruments on a sterile table. The foot and ankle were disinfected again and new sterile draping was applied. Using standard anteromedial and anterolateral portals, an anterior ankle arthroscopy was performed with the patient in the supine position. Alternatively, posterior ankle arthroscopy was performed with the patient in the prone position through posteromedial and posterolateral portals utilizing the technique described by Van Dijk et al (14). In patients in whom a combined posterior and anterior ankle arthroscopy was planned, the posterior procedure was used initially, followed by the anterior procedure. If excision of an os peroneum was required, it was always done with the patient in the same position, by making an incision over the lateral aspect of the foot in line with the peroneal tendons.

For the patients in whom peroneal tendoscopy was performed as an independent procedure, postoperative management included partial weight bearing on the operated foot with the aid of two crutches for the first two weeks, and active movements (flexion-extension of the ankle) were encouraged from the first day. For the patients in whom peroneal tendoscopy was performed as an accessory procedure, the postoperative management depended on the type of pathology that was treated.

We found 3 peroneus brevis tendon partial tears, 4 cases of a low-lying peroneus brevis muscle belly, 5 cases of tenosynovitis, and 1 case of an intrasheath subluxation (Table 1). During tendoscopy, we performed tenosynovectomy in cases with tenosynovitis, we cut off the redundant distal muscle fibers in cases with a low-lying peroneus brevis muscle belly with a shaver and radiofrequency probe, and for peroneus brevis tendon partial tears we performed tendoscopic debridement. In 5 of the 13 patients, we performed peroneal tendoscopy as a solitary procedure and in 8 patients as an accessory procedure together with anterior ankle arthroscopy (3 cases), posterior ankle arthroscopy (1 case), combined posterior and anterior ankle arthroscopy (2 cases), and open surgery for excision of the os peroneum (2 cases). For treatment of a degenerative tear of the peroneus brevis tendon (patient No. 10) after tendoscopic debridement, we performed a mini-open repair with tubularization of the tendon. There were no perioperative complications and the rehabilitation period was uneventful. At one-year follow-up, all patients were satisfied with the final outcome related to tendoscopic procedure and had no pain or clicking sensations.

**Table 1:** List of patients with their clinical presentation and history of injury together with final endoscopic finding and treatment procedure of choice. Depending on the diagnosis, peroneal tendoscopy was performed as a solitary or an accessory procedure*

Patient number	Age/sex	Clinical presentation	Previous injury	Endoscopic findings	Endoscopic treatment	Accessory procedure
1	46/F	retrofibular tenderness	ankle inversion	significant tenosynovitis	debridement of synovitis	no
2	47/F	retrofibular pain, crepitus	no	longitudinal tear of PB tendon	debridement of rupture	no
3	32/F	retrofibular tenderness with swelling, anteromedial ankle pain	no	low-lying muscle belly of PB tendon	resection of low-lying muscle belly	anterior ankle arthroscopy with debridement and microfracturing of the posteromedial OCD lesion of the talus
4	48/F	retrofibular tenderness along the PL tendon distal to the fibula, radiographically proven os peroneum, lateral plantar foot pain	ankle inversion	no significant tenosynovitis	debridement of synovitis	open excision of os peroneum
5	13/M	retrofibular pain with palpable and visible clicking during active eversion and dorsiflexion of the foot and ankle	no	low-lying muscle belly of PB tendon	resection of low-lying muscle belly	no
6	27/F	pain present in retrofibular and anterolateral part of the ankle	ankle inversion	low-lying muscle belly of PB tendon	resection of low-lying muscle belly	anterior ankle arthroscopy with debridement and microfracturing of the anterolateral OCD lesion of the talus
7	20/M	retrofibular tenderness with swelling	ankle inversion	significant tenosynovitis	debridement of synovitis	no
8	26/M	retrofibular tenderness with swelling of the ankle with decreased ROM	no	loose bodies with significant tenosynovitis	removal of loose bodies with debridement of synovitis	posterior and anterior ankle arthroscopy with complete synoviectomy and removal of loose bodies
9	31/M	retrofibular pain, posterior ankle pain	ankle inversion	low-lying muscle belly of PB tendon and longitudinal tear of PB tendon	resection of the low-lying PB muscle belly and rupture debridement	posterior ankle arthroscopy with a resection of a prominent posterior talar process
10	30/M	retrofibular tenderness with swelling	ankle contusion	significant tenosynovitis and longitudinal tear of PB tendon	debridement of synovitis, mini-open repair and tubularization of the tendon	no
11	58/F	retrofibular tenderness, lateral plantar foot pain, radiographically proven os peroneum	ankle inversion	no significant tenosynovitis	debridement of synovitis	open excision of os peroneum
12	15/F	retrofibular pain, anterolateral ankle pain and tenderness in the anterolateral ankle gutter	ankle inversion	low-lying muscle belly of PB tendon	resection of low-lying muscle belly	anterior ankle arthroscopy with debridement of soft-tissue impingement lesion
13	24/M	retrofibular tenderness with swelling, anterior et posterior ankle pain	no	low-lying muscle belly of PB tendon	resection of low-lying muscle belly	posterior and anterior ankle arthroscopy with debridement and resection of impinging osteophytes

*PB – peroneus brevis muscle; OCD – osteochondritis dissecans; PL – peroneus longus muscle; ROM – range of motion.

## Discussion

Peroneal tendoscopy can endoscopically solve many peroneal tendon disorders while preserving the integrity of the superior peroneal retinaculum (2,10-13). The main indication for this procedure is persistent posterolateral ankle pain (3). Peroneal tendoscopy was initially proposed and described as a solitary procedure for treatment of peroneal tendons disorders (2). However, peroneal tendons disorders are often associated with, and appear secondary to intra- or extra-articular ankle pathology such as lateral ankle instability, distal fibula fractures, anterior or posterior impingement of the ankle, chondral or osteochondral lesions of the talus, or subtalar malalignment (1,10-13). The common symptom in all these patients is posterolateral ankle pain. Therefore, treatment of these combined disorders often requires more than one operative procedure and in these situations peroneal tendoscopy serves as an accessory procedure, conjoined with eg, ankle arthroscopy, anatomic repair, or reconstruction of the lateral ankle ligaments, and various other procedures (1). Our clinical experience confirmed that peroneal tendoscopy served as a valuable adjunct to other procedures that were performed for treatment of ankle pain. This is also supported by the fact that peroneal tendons and the ankle form a single functional unit. In this manner, the whole functional unit is treated in a single stage procedure, solving all present disorders and providing a proper substrate for the physical rehabilitation that follows such procedure. Additionally, combined procedures reduce the rehabilitation period, ie, off-the-work period. This is extremely important for elite athletes, since the off-training period has a significant influence on their top performance level.

We have to emphasize that the diagnosis of posterolateral ankle pain is frequently very challenging since clinical tests and imaging techniques like MRI are often inconclusive. In severe cases, where frank peroneal tendon tears and severe tenosynovitis are present, MRI serves as a powerful diagnostic tool. However, MRI findings can sometimes be confusing, especially in more subtle cases, still associated with significant symptoms. Relatively frequent anatomic variations of the peroneal tendons were identified as a predisposing factor for peroneal tendon disorders (a low-lying peroneus brevis muscle belly, an accessory peroneus quartus muscle, hypertrophy of peroneal tubercle, a shallow or narrow retromalleolar groove, and the presence of an os peroneum) (15-21) (Table 2). Therefore, it is important to be familiar with these anatomic variations and to take them into consideration when making a diagnosis or decision to perform surgical treatment (22-26).

**Table 2:** Definition and incidence of peroneal tendons' anatomic variations and potential disorders they may cause*

Anatomic variation	Definition	Incidence	Cause	Predispose to
Low-lying PB muscle belly	an anomalous extension of the PB muscle into and distal to the fibular groove	not available in literature	crowding of the retromalleolar groove and stretching of the SPR	PB tendon tear, tenosynovitis, intrasheath subluxation, peroneal dislocation
Peroneus quartus muscle	commonly originates from the muscular portion of the PB muscle in the distal third of the lower-leg and inserts in the retrotrochlear eminence of the calcaneus	6.6 to 21.7% (15,16)	crowding of the retromalleolar groove and stretching of the SPR	PB tendon tear, tenosynovitis, intrasheath subluxation, peroneal dislocation
Hypertrophy of peroneal tubercle	peroneal tubercle, a bony protuberance located at the lateral aspect of the calcaneus separates PL tendon from PB tendon and is considered hypertrophied if higher than 5 mm (17)	presence of peroneal tubercle varies from 24 to 98.58%; in one-third of those individuals, peroneal tubercle hypertrophy exists (18)	increases mechanical stress on peroneal tendons and causes mechanical irritation of the tendons	tenosynovitis, tendon tear
Shallow or narrow retromalleolar groove	the lack of concavity of the posterior distal fibula, commonly there is a sulcus in the posterior aspect of the distal fibula with a width of 5 to 10 mm and a depth of up to 3 mm (12)	18% (19)	may affect the stability of the peroneal tendons	tenosynovitis, intrasheath subluxation, peroneal dislocation
Os peroneum	an oval or round accessory ossicle located within the substance of the PL tendon at the level of the calcaneocuboid joint. It can be found in cartilaginous, fibrocartilagenous or ossified forms	when ossified, it is visible on 4.7%-31.7% of foot radiographs, usually unilaterally (20)	in certain cases (os peroneum fracture or a diastases of a multipartite os peroneum) might actually be responsible for tendon damage and fatigue	POPS, tenosynovitis, tendon tear

*PB – peroneus brevis muscle; SPR – superior peroneal retinaculum; PL – peroneus longus muscle; POPS – painful os peroneum syndrome.

Peroneal tendon disorders have been diagnosed in as many as 25% of patients with ankle instability (27). This is why peroneal tendoscopy has been suggested as an obligatory surgical procedure prior to lateral ligament reconstruction (4). In our opinion, despite the large number of cases, asymptomatic patients should not be compelled to undergo peroneal tendoscopy as an accessory procedure prior to ankle stabilization.

Peroneal tendoscopy is a technically demanding procedure that requires the skill of an experienced arthroscopist and excellent knowledge of regional anatomy in order to avoid complications. Potential complications include peroneal tendon injury, damage to the sural nerve or the communicating branch of the sural nerve to the superficial peroneal nerve. These structures are especially at risk while creating the portals. The most common pitfall of tendoscopy is the rupture of the tendon sheath during the passage of surgical instruments, which causes a visual impairment due to the collapse of the sheath and extravasations of the fluid (28). The use of a Wissinger rod for accurate portal exchanging of the scope will reduce the risk of extravasation and edema.

In conclusion, peroneal tendoscopy is a useful procedure with low morbidity and excellent functional results to treat miscellaneous peroneal tendon disorders. In our series of patients it was a good adjunct to other procedures performed for posterolateral ankle pain. Therefore, we would like to emphasize that peroneal tendoscopy can have a dual role in the orthopaedic armamentarium, ie, as a solitary procedure for certain indications, but also as a valuable accessory procedure.

## References

[1] Marmotti A, Cravino M, Germano M, Del Din R, Rossi R, Tron A (2012). Peroneal tendoscopy.. Curr Rev Musculoskelet Med.

[2] Van Dijk CN, Kort N (1998). Tendoscopy of the peroneal tendons.. Arthroscopy.

[3] Scholten PE, Dijk CN (2006). Tendoscopy of the peroneal tendons.. Foot Ankle Clin.

[4] Sammarco VJ (2009). Peroneal tendoscopy: indications and techniques.. Sports Med Arthrosc.

[5] Vega J, Golano P, Batista JP, Malagelada F, Pellegrino A (2013). Tendoscopic procedure assoiciated with peroneal tendons.. Tech Foot & Ankle..

[6] Vega J, Cabestany JM, Golano P, Perez-Carro L (2008). Endoscopic treatment for chronic Achilles tendinopathy.. Foot Ankle Surg.

[7] Steenstra F, van Dijk CN (2006). Achilles tendoscopy.. Foot Ankle Clin..

[8] Reilingh ML, de Leeuw PAJ, van Sterkenburg MN, van Dijk CN (2010). Tendoscopy of posterior tibial and peroneal tendons.. Tech Foot & Ankle..

[9] Chow HT, Chan KB, Lui TH (2005). Tendoscopic debridement for stage I posterior tibial tendon dysfunction.. Knee Surg Sports Traumatol Arthrosc.

[10] Selmani E, Gjata V, Gjika E (2006). Current concepts review: peroneal tendon disorders.. Foot Ankle Int.

[11] Heckman DS, Reddy S, Pedowitz D, Wapner KL, Parekh SG (2008). Operative treatment for peroneal tendon disorders.. J Bone Joint Surg Am.

[12] Philbin TM, Landis GS, Smith B (2009). Peroneal tendon injuries.. J Am Acad Orthop Surg.

[13] Heckman DS, Gluck GS, Parekh SG (2009). Tendon disorders of the foot and ankle, part 1: peroneal tendon disorders.. Am J Sports Med.

[14] Van Dijk CN, Scholten PE, Krips R (2000). A 2-portal endoscopic approach for diagnosis and treatment of posterior ankle pathology.. Arthroscopy.

[15] Zammit J, Singh D (2003). The peroneus quartus muscle. Anatomy and clinical relevance.. J Bone Joint Surg Br.

[16] Sobel M, Levy ME, Bohne WH (1990). Congenital variations of the peroneus quartus muscle: an anatomic study.. Foot Ankle.

[17] Saupe N, Mengiardi B, Pfirrmann CW, Vienne P, Seifert B, Zanetti M (2007). Anatomic variants associated with peroneal tendon disorders: MR imaging findings in volunteers with asymptomatic ankles.. Radiology.

[18] Hyer CF, Dawson JM, Philbin TM, Berlet GC, Lee TH (2005). The peroneal tubercle: description, classification, and relevance to peroneus longus tendon pathology.. Foot Ankle Int.

[19] Wang XT, Rosenberg ZS, Mechlin MB, Schweitzer ME (2005). Normal variants and diseases of the peroneal tendons and superior peroneal retinaculum: MR imaging features.. Radiographics.

[20] Kose O (2012). The accessory ossicles of the foot and ankle; a diagnostic pitfall in emergency department in context of foot and ankle trauma.. JAEM.

[21] Lee SJ, Jacobson JA, Kim SM, Fessell D, Jiang Y, Dong Q (2013). Ultrasound and MRI of the peroneal tendons and associated pathology.. Skeletal Radiol.

[22] Ho KK, Chan KB, Lui TH, Chow YY (2013). Tendoscopic-assisted repair of complete rupture of the peroneus longus associated with displaced fracture of the os peroneum–case report.. Foot Ankle Int.

[23] Kassim MM, Rosenfeld P (2012). Tendoscopic debridement of peroneus quartus muscle for chronic lateral ankle pain: a case report.. Foot Ankle Int.

[24] Lui TH (2012). Tendoscopic resection of low-lying muscle belly of peroneus brevis or quartus.. Foot Ankle Int.

[25] Vega J, Golano P, Dalmau A, Viladot R (2011). Tendoscopic treatment of intrasheath subluxation of the peroneal tendons.. Foot Ankle Int.

[26] Michels F, Jambou S, Guillo S, Van Der Bauwhede J (2013). Endoscopic treatment of intrasheath peroneal tendon subluxation. Case Rep Med.

[27] DiGiovanni BF, Fraga CJ, Cohen BE, Shereff MJ (2000). Associated injuries found in chronic lateral ankle instability.. Foot Ankle Int.

[28] Panchbhavi VK, Trevino SG (2003). The technique of peroneal tendoscopy and its role in management of peroneal tendon anomalies.. Tech Foot & Ankle..

